# Comorbidities and use of analgesics in people with knee pain: a study in the Nottingham Knee Pain and Health in the Community (KPIC) cohort

**DOI:** 10.1093/rap/rkac049

**Published:** 2022-06-15

**Authors:** Subhashisa Swain, Gwen Sascha Fernandes, Aliya Sarmanova, Ana M Valdes, David A Walsh, Carol Coupland, Michael Doherty, Weiya Zhang

**Affiliations:** Academic Rheumatology, School of Medicine, University of Nottingham, Nottingham City Hospital; Pain Centre Versus Arthritis, University of Nottingham; NIHR Nottingham Biomedical Research Centre, Nottingham; Nuffield Department of Primary Care Health Sciences, University of Oxford, Oxford; Population Health Sciences, Bristol Medical School, University of Bristol, Bristol; Population Health Sciences, Bristol Medical School, University of Bristol, Bristol; Academic Rheumatology, School of Medicine, University of Nottingham, Nottingham City Hospital; Pain Centre Versus Arthritis, University of Nottingham; NIHR Nottingham Biomedical Research Centre, Nottingham; Academic Rheumatology, School of Medicine, University of Nottingham, Nottingham City Hospital; Pain Centre Versus Arthritis, University of Nottingham; NIHR Nottingham Biomedical Research Centre, Nottingham; Centre for Academic Primary Care, School of Medicine, University of Nottingham, Nottingham, UK; Academic Rheumatology, School of Medicine, University of Nottingham, Nottingham City Hospital; Pain Centre Versus Arthritis, University of Nottingham; NIHR Nottingham Biomedical Research Centre, Nottingham; Academic Rheumatology, School of Medicine, University of Nottingham, Nottingham City Hospital; Pain Centre Versus Arthritis, University of Nottingham; NIHR Nottingham Biomedical Research Centre, Nottingham

**Keywords:** OA, comorbidity, community survey, NSAIDs, paracetamol, opioids, interaction

## Abstract

**Objectives:**

The aims were to examine the prevalence of comorbidities and role of oral analgesic use in people with knee pain (KP) compared with those without.

**Methods:**

The Knee Pain and related health In the Community (KPIC) cohort comprises community-derived adults aged ≥40 years, irrespective of knee pain. Thirty-six comorbidities across 10 systems were compared between people with KP and controls without KP or knee OA. Multivariable logistic regression analysis was used to determine the adjusted odds ratio (aOR) and 95% CI for multimorbidity (at least two chronic conditions) and each specific comorbidity. Both prescribed and over-the-counter analgesics were included in the model, and their interactions with KP for comorbidity outcomes were examined.

**Results:**

Two thousand eight hundred and thirty-two cases with KP and 2518 controls were selected from 9506 baseline participants. The mean age of KP cases was 62.2 years, and 57% were women. Overall, 29% of the total study population had multimorbidity (KP cases 34.4%; controls 23.8%). After adjustment for age, sex, BMI and analgesic use, KP was significantly associated with multimorbidity (aOR 1.35; 95% CI 1.17, 1.56) and with cardiovascular (aOR 1.25; 95% CI 1.08, 1.44), gastrointestinal (aOR 1.34; 95% CI 1.04, 1.92), chronic widespread pain (aOR 1.54; 95% CI 1.29, 1.86) and neurological (aOR 1.32; 95% CI 1.01, 1.76) comorbidities. For multimorbidity, the use of paracetamol and opioids interacted positively with KP, whereas the use of NSAIDs interacted negatively for seven comorbidities.

**Conclusion:**

People with KP are more likely to have other chronic conditions. The long-term benefits and harms of this change remain to be investigated.

**Trial registration:**

ClinicalTrials.gov, http://clinicaltrials.gov, NCT02098070.

Key messages

- People with knee pain have increased association with multiple chronic conditions and other comorbidities.
- Use of paracetamol and opioids for knee pain is strongly associated with other chronic conditions.
- However, association of the use of NSAIDs with comorbidities was less evident.

## Introduction

Knee pain (KP) is one of the major reported musculoskeletal problems worldwide [1], and in middle-aged and older people it is commonly associated with knee OA [2]. According to a recent systematic review, one in five people aged ≥40 years around the world have knee OA [3]. The burden of KP increases with age and contributes to functional decline and disability [4–6]. Like KP, many other chronic conditions are more likely to occur in later life [5–8].

A recent systematic review reported that 67% of people with any OA have at least one other chronic condition [9]. Comorbidity (the presence of any other condition with an index disease) research in KP has focused mainly on chronic widespread pain [10] or psychological problems [11]; however, recent studies have shown that the risks of cardiovascular disease, diabetes, upper gastrointestinal and psychological conditions also are increased in people with KP or OA [12, 13]. This is supported by a retrospective cohort study in the UK in people aged >20 years, which reported that 53% of people with OA had at least two comorbidities at the time of OA diagnosis [14]. Comorbidities in KP are associated with worse pain, more physical disability [15] and increased risk of mortality [16, 17]. Also, they can influence treatment options, both pharmacological and non-pharmacological, and complicate shared management decision-making [18–20]. The increased number of comorbidities in KP can also influence the choice and outcomes of knee replacement surgery [21].

Ageing, obesity, movement restriction, pathophysiological changes and analgesic drug intake might all contribute to development of comorbidities in KP. However, current evidence is often prone to selection bias [9]. For example, medical record-based studies do not capture individuals in the general population who do not consult for health-care advice and also lack information on use of non-prescribed medications, such as over-the-counter (self-purchased) analgesics [22]. Such information is crucial in understanding the true burden of comorbidities in KP. Therefore, we used community survey data to explore comorbidities in KP. The aims of this study were to identify the association of KP with other chronic conditions and to examine the use of oral analgesics in people with KP and comorbidities.

## Methods

We used baseline data from the Nottingham Knee Pain and related health In the Community (KPIC) study, a prospective cohort study in the East Midlands region of the UK, which began in 2014 [23]. The study was approved by the Nottingham Research Ethics Committee 1 (NREC reference 14/EM/0015) and registered with ClinicalTrials.gov (NCT02098070). All written informed consent forms were stored safely and securely in Academic Rheumatology, Nottingham City Hospital.

### Study design and population

This was a cross-sectional study using baseline questionnaire data of KPIC. At baseline, 40 505 questionnaires were mailed to all men and women aged ≥40 years, irrespective of knee pain status, who were registered with 12 participating general practices in the East Midlands area. Exclusion criteria were inability to give informed consent, terminal illness or severe mental illness [18]. The questionnaire was accompanied by a covering letter from each person’s general practitioner introducing the study and its objectives. Return of a completed questionnaire in a pre-paid envelope to Academic Rheumatology (City Hospital, Nottingham) was taken as implicit consent. Completed questionnaires were returned by 9506 individuals (23% response rate). Full details of the KPIC study have been published elsewhere [23].

For the present study, KP was defined as either chronic knee pain or physician-diagnosed knee OA (KOA). KP was defined using a self-reported question: ‘Have you experienced pain in or around a knee on most days of the last month and has your current knee pain lasted more than 3 months?’. Clinically, chronic KP in people >40 years old has shown congruity with KOA [24]. KOA was defined as any person reporting physician-diagnosed KOA who answered, ‘Yes’ to the question: ‘Have you ever been diagnosed by your doctor as having osteoarthritis of the knee?’. Individuals were required to have positive responses to either of these two questions for inclusion in the KP/KOA case group. The reason that KPIC focused on knee pain rather than OA is because knee pain is the malady, and radiographic OA is a risk factor for pain rather than an important disease. The findings are likely to be relevant to KOA, given that most chronic knee pain in people >40 years old is associated with OA.

We selected all the eligible people with neither KP nor KOA from the KPIC database as controls. Of 9506 individuals who responded to the survey, 78 gave no information on age or sex, and 4078 people gave no information about KP/KOA, and these were excluded from the analyses. Thus, the total available eligible individuals for the study were 5350, of whom 2832 had either KP and/or KOA and the remaining 2518 were selected as controls. Controls were not matched for any variables. Details of the participant selection is provided in Supplementary Fig. S1, available at *Rheumatology Advances in Practice* online.

### Sample size

We used the available information from a previous study on comorbidities in OA for sample size calculation [25]. The minimum needed to detect an odds ratio (OR) of 1.36 with 42.2% controls exposed with a power of 80% and significance level of 0.05 was 1790 (1:1 ratio).

### Comorbidities

KPIC also collected information on self-reported physician-diagnosed chronic conditions. The questionnaire provided a check list of 10 physician-diagnosed chronic conditions to choose from [high cholesterol, heart attack/angina, high blood pressure, OA, diabetes, stroke, irritable bowel syndrome (IBS), FM, chronic fatigue syndrome and cancer], together with an open-ended question ‘other’. From open responses to the ‘others’, 26 additional chronic conditions were added to the above list of 10, giving a total of 36 possible comorbidities. These were grouped into 10 systems according to similar physiological and pathological characteristics [cardiovascular, gastrointestinal, chronic widespread pain, musculoskeletal, psychological, genitourinary, respiratory, cancer (any), neurological and endocrine] recorded in primary care records [10]. Details of the conditions and their grouping are given in (Supplementary Appendix 1, available at *Rheumatology Advances in Practice* online).

### Analgesics

Self-reported use of prescribed and/or over-the-counter oral analgesics at baseline, specifically paracetamol, NSAIDs including Cyclooxygenase-2 (COX-2) selective inhibitors, and opioids (might or might not be for KP), were included in the analysis. Participants were asked to list the names of current medicines and the duration of their use in years and months. Use of drugs by the participants was coded as ‘yes’, otherwise ‘no’. Furthermore, the years of exposure were categorized as 0, 1–5, 6–10 or >10 years since the first date of reported use.

### Data analysis

Descriptive analysis was done for baseline characteristics such as age, sex,and BMI. Categorical variables were described with proportions, and continuous variables were described as the mean and s.d. or median with interquartile range (IQR) as appropriate across the KP and non-KP groups. Obesity was defined as a BMI of ≥30 kg/m^2^ [26]. Univariate associations were explored through χ^2^ tests and unpaired Student’s *t*-tests. Participants with missing information on age and sex were excluded from the final analysis [27].

The main objective was to examine the prevalence of any comorbidities in those with KP compared with those without. The number of comorbidities was calculated by adding all the chronic conditions reported (other than KP) and grouped into categories of none, one, two or three or more. The prevalence with individual comorbidities in the two groups was also calculated. Also, the pattern of combinations of two chronic conditions (dyads) was described and examined using simple exploratory methods. We did not explore patterns in more than two conditions owing to small numbers of people in each possible pattern. In this analysis, we created a network mapping of conditions in relationship to each other and their connection to obesity. The leading combination of two comorbidities within each of the two groups was plotted.

Associations between individual comorbidities, multimorbidity (at least two comorbidities) (yes/no) and KP (yes/no) were estimated in a comparison of KP cases with the controls using univariate and multivariable logistic regression models [28]. In the multivariable logistic regression model, initial adjustment was for age, sex, BMI and other comorbidities, and secondarily for NSAIDs, opioids and paracetamol. Both multiplicative and additive interactions between each of the analgesics (NSAIDs, opioids and paracetamol) with KP for each comorbidity outcome were examined in turn. Interaction on an additive scale means that the combined effect of two exposures is larger (or smaller) than the sum of the individual effects of the two exposures, whereas interaction on a multiplicative scale means that the combined effect is larger (or smaller) than the product of the individual effects. We estimated two parameters of the additive interaction, such as relative excessive risk attributable to interaction (RERI), and attributable proportion (AP) to test the consistency of additive interaction [29]. Details of the methods and terms used are given in (Supplementary Appendix 2, available at *Rheumatology Advances in Practice* online). We also estimated the dose–response relationship between the years of exposure to analgesics and association with multimorbidity, and a trend test was performed for each type of analgesic. Adjustment for multiple testing was done using the false discovery rate method suggested by Benjamini & Yekutieli [30] using the FDR package in R. To visualize comorbidity connections, disease network maps were constructed for both groups. The figures were made with the Igraph R package v.1.0.1 [31]. All other analyses were conducted using Stata SE v.15 (StataCorp, College Station, TX, USA) and R (v.3.5).

## Results

**Fig. 1:**
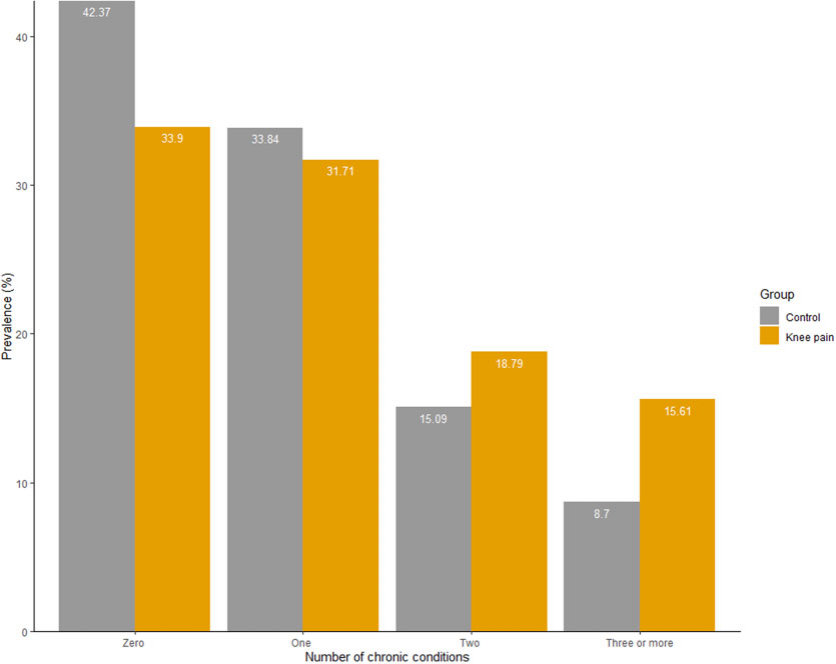
Distribution of the number of chronic conditions in both knee pain and non-knee pain groups (χ^2^ test *P*-value <0.05)

Of the 9506 study participants, 1674 (17.6%) reported KP only, 370 (3.9%) reported physician-diagnosed KOA only, and 909 (9.6%) reported both. In total, 2953 (31.1%) had KP as defined for this study, of whom 2832 had complete demographic data and were included. The mean age was 62 years (s.d. 10.4 years), and 58% were women. In the 2518 non-KP controls, the mean age was 61 years (s.d. 11 years), and 55% were women. The distribution of BMI and other covariates across the groups are given in Table 1. The prevalence of obesity was significantly higher in KP cases (33.0%) than controls (22.2%). Use of oral NSAIDs, opioids and paracetamol were significantly higher in people with KP (Table 1). Of the 36 comorbidities considered, the median number of chronic conditions (excluding KP) was higher in people with KP (1, IQR 0–2) than in non-KP controls (1, IQR 0–1; *P* < 0.05). The prevalence of a single morbidity was 31.7 and 33.8% in the KP and non-KP groups, respectively, whereas prevalence of multimorbidity was higher in the KP (34%) than the control (24%) group (Fig. 1).

**Table 1:** Characteristics of study participants

Characteristic	Cases (KP) (*n* = 2832)	Controls (non-KP) (*n* = 2518)	*P*-value*
Age^a^, mean (s.d.), years	62.38 (10.4)	60.98 (10.6)	0.22
Female, *n* (%)	1663 (58.7)	1392 (55.3)	0.011
BMI^b^, mean (s.d.), kg/m^2^	29.01 (6.1)	27.09 (5.0)	<0.001
Obesity, *n* (%)	934 (33.0)	535 (22.2)	<0.001
NSAID users, *n* (%)	479 (16.9)	171 (6.8)	<0.001
Opioid users, *n* (%)	562 (19.9)	163 (6.5)	<0.001
Paracetamol users, *n* (%)	588 (20.8)	204 (8.1)	<0.001
Number of additional chronic conditions, mean (s.d.)	1.25 (1.3)	0.93 (1.1)	–
Number of additional chronic conditions, median (IQR)	1 (0–2)	1 (0–1)	<0.001

a Age as of 1 January 2015.

b BMI data were available for 5126 participants (KP: 2420; non-KP: 2706).

* For age and BMI, Student’s *t*-test was performed; for median number of comorbidities, the Mann–Whitney *U*-test; and for the remaining covariates the χ^2^ test.

IQR: interquartile range; KP: knee pain.

Of the 36 individual conditions that were considered, 13 were significantly more prevalent in the KP group than in controls (Table 2). FM [adjusted odds ratio (aOR) 3.06; 95% CI 2.10, 4.47], upper gastrointestinal problems (aOR 2.17; 95% CI 1.34, 3.52), vision problems (aOR 2.14; 95% CI 1.07, 4.29), chronic fatigue syndrome (aOR 1.71; 95% CI 1.07, 2.72), IBS (aOR 1.50; 95% CI 1.25, 1.80), diabetes (aOR 1.36; 95% CI 1.12, 1.70), heart disease (aOR 1.38; 95% CI 1.12, 1.70), hypertension (aOR 1.32; 95% CI 1.16, 1.50) and high cholesterol (aOR 1.14; 95% CI 1.01, 1.30) were significantly associated with KP in multivariable models including adjustment for age, sex, BMI, other comorbidities and analgesic drug use. The prevalence and association of system-specific comorbidities with each group are given in Table 2. The odds of having multimorbidity was 1.35 times (95% CI 1.17, 1.56) higher in people with KP. Out of 10 system-specific comorbidities, 4 had a significant association with KP after adjusting for age, sex, BMI, hyperlipidaemia, other comorbidities and analgesics. The risks of having cardiovascular (aOR 1.25; 95% CI 1.08, 1.44), gastrointestinal (aOR 1.34; 95% CI 1.04, 1.92), chronic widespread pain (aOR 1.54; 95% CI 1.29, 1.86) and neurological (aOR 1.32; 95% CI 1.01, 1.76) comorbidities were significantly higher in people with KP (Table 3).

**Table 2:** Prevalence and odds ratio of comorbidities between people with and without knee pain

Comorbidity	KP (*n* = 2832), * n* (%)	Non-KP (*n* = 2518), * n* (%)	Unadjusted OR (95% CI)	Adjusted OR (95% CI)	
				Model 1^a^	Model 2^b^
Multimorbidity	974 (34.4)	599 (23.8)	1.68 (1.49, 1.89)*	–	1.35 (1.17, 1.56)*
Cardiovascular	1148 (59.5)	789 (31.3)			
Hypertension	1028 (36.3)	707 (28.1)	1.46 (1.30, 1.64)*	1.39 (1.24, 1.58)*	1.32 (1.16, 1.50)*
Heart disease	282 (10.0)	182 (7.2)	1.42 (1.17, 1.72)*	1.39 (1.14, 1.70)*	1.38 (1.12, 1.70)*
Endocrine	450 (15.9)	334 (13.3)			
Diabetes	365 (12.9)	232 (9.2)	1.46 (1.22, 1.73)*	1.43 (1.19, 1.70)*	1.36 (1.13, 1.64)*
Thyroid	105 (3.7)	108 (4.3)	0.86 (0.65, 1.13)	0.80 (0.60, 1.06)	0.88 (0.66, 1.17)
Gastrointestinal	109 (3.8)	65 (2.6)			
Upper gastrointestinal	58 (2.1)	25 (1.0)	2.08 (1.30, 3.34)*	2.00 (1.25, 3.22)*	2.17 (1.34, 3.52)*
Colitis	26 (0.9)	17 (0.7)	1.36 (0.74, 2.51)	1.37 (0.74, 2.53)	1.27 (0.67, 2.40)
Liver and gall bladder	17 (0.6)	17 (0.7)	0.89 (0.45, 1.74)	0.83 (0.42, 1.63)	0.76 (0.38, 1.54)
Coeliac disease	12 (0.4)	6 (0.2)	1.78 (0.67, 4.75)	1.58 (0.59, 4.22)	1.40 (0.51, 3.88)
Cancer	239 (8.4)	226 (8.9)	0.93 (0.77, 1.13)	0.90 (0.71, 1.12)	0.82 (0.67, 1.00)
Chronic widespread pain	564 (19.9)	306 (12.1)			
Fibromyalgia (FM)	152 (5.4)	37 (1.5)	3.80 (2.64, 5.46)*	3.83 (2.65, 5.52)*	3.06 (2.10, 4.47)*
Back pain	63 (2.2)	35 (1.4)	1.63 (1.06, 2.44)*	1.62 (1.06, 2.46)	1.03 (0.66, 1.60)
Chronic fatigue syndrome	62 (2.2)	29 (1.1)	1.92 (1.23, 2.99)*	1.95 (1.25, 3.05)*	1.71(1.07, 2.72)*
Migraine	20 (0.7)	21 (0.8)	0.85 (0.46, 1.56)	0.88 (0.47, 1.63)	0.67 (0.35, 1.28)
Irritable bowel syndrome	401 (14.2)	225 (8.9)	1.68 (1.41, 1.99)*	1.65 (1.39, 1.96)*	1.50 (1.25, 1.80)*
Neurological	188 (6.6)	113 (4.5)			
Stroke	105 (3.7)	65 (2.6)	1.45 (1.06, 1.99)*	1.36 (0.98, 1.86)	1.30 (0.93, 1.79)
Neurological (other)	39 (1.4)	16 (0.6)	2.18 (1.21, 3.91)*	2.18 (1.21, 3.93)	1.67 (0.91, 3.07)
Epilepsy	20 (0.7)	9 (0.4)	1.98 (0.90, 4.36)	1.74 (0.76, 3.97)	2.07 (0.94, 4.57)
Parkinson’s disease	10 (0.4)	5 (0.2)	1.78 (0.61, 5.21)	1.66 (0.57, 4.89)	1.77 (0.59, 5.35)
Respiratory (COPD)	171 (6.0)	131 (5.2)	1.17 (0.92, 1.47)	1.16 (0.91, 1.47)	1.12 (0.88, 1.43)
Musculoskeletal	119 (4.2)	96 (3.8)			
Osteoporosis	36 (1.3)	27 (1.1)	1.19 (0.71, 1.96)	1.07 (0.64, 1.77)	1.04 (0.61, 1.74)
Rheumatoid arthritis	32 (1.1)	30 (1.2)	0.95 (0.57, 1.56)	0.92 (0.56, 1.52)	0.69 (0.41, 1.17)
Gout	28 (1.0)	21 (0.8)	1.18 (0.67, 2.09)	1.21 (0.69, 2.15)	1.19 (0.66, 2.16)
SLE	6 (0.2)	4 (0.2)	1.33 (0.38, 4.73)	1.43 (0.40, 5.11)	1.02 (0.44, 2.36)
Psoriatic Arthritis	7 (0.3)	3 (0.1)	2.08 (0.54, 8.04)	2.29 (0.59, 8.91)	2.08 (0.51, 8.42)
Polymyalgia	13 (0.5)	12 (0.5)	0.96 (0.44, 2.11)	0.85 (0.39, 1.88)	0.53 (0.23, 1.22)
Psychological problem	90 (3.2)	58 (2.3)	1.39 (0.99, 1.94)	1.28 (0.86, 1.92)	1.25 (0.88, 2.00)
Genitourinary	50 (1.8)	39 (1.5)			
Prostate problem	23 (0.8)	23 (0.9)	0.89 (0.50, 1.58)	0.93 (0.52, 1.67)	1.00 (0.54, 1.82)
Renal problem	27 (0.9)	16 (0.6)	1.50 (0.81, 2.80)	1.47 (0.79, 2.75)	1.47 (0.77, 2.80)
Others					
High cholesterol	900 (31.8)	674 (26.8)	1.27 (1.13, 1.43)*	1.22 (1.08, 1.38)*	1.14 (1.01, 1.30)*
Vision problem	279 (0.9)	12 (0.5)	2.01 (1.02, 3.97)*	1.83 (0.92, 3.63)	2.14 (1.07, 4.29)*
Peripheral vascular disease	20 (0.7)	12 (0.5)	1.48 (0.72, 3.04)	1.52 (0.74, 3.12)	1.69 (0.79, 3.63)
Psoriasis	19 (0.7)	13 (0.5)	1.30 (0.64, 2.64)	1.32 (0.65, 2.68)	1.28 (0.61, 2.66)
Sleep problem	13 (0.5)	11 (0.4)	1.05 (0.47, 2.35)	1.13 (0.51, 2.53)	1.02 (0.44, 2.36)
Hearing problem	10 (0.4)	7 (0.3)	1.27 (0.48, 3.34)	1.19 (0.45, 3.15)	1.09 (0.40, 2.96)
Skin disease	7 (0.3)	9 (0.4)	0.69 (0.26, 1.86)	0.75 (0.28, 2.02)	0.82 (0.29, 2.27)
Lymphatic problem	6 (0.2)	4 (0.2)	1.33 (0.38, 4.73)	1.25 (0.35, 4.44)	0.93 (0.25, 3.48)

^a^ Adjusted for age, sex and BMI.

^b^ Adjusted for age, sex, BMI, opioids, NSAIDs and paracetamol.

* *P*-value < 0.05 (adjusted for multiple testing using the Benjamin–Hochberg method).

COPD: chronic obstructive pulmonary disease; OR: odds ratio; SLE: Systemic Lupus Erythematosus.

**Table 3:** Association of prescription of analgesics with comorbidities

Comorbidity			Paracetamol		Opioids			NSAIDs		
	aOR^a^ (95% CI)	Multiplicative Interaction aOR^a^ (95% CI)	RERI (95% CI)^a^	Attributable proportion (95% CI)^a^	Multiplicative Interaction aOR^a^ (95% CI)	RERI (95% CI)^a^	Attributable proportion (95% CI)^a^	Multiplicative Interaction aOR^a^ (95% CI)	RERI (95% CI)^a^	Attributable proportion (95% CI)^a^
Multimorbidity	**1.35 (1.17, 1.56)**	2.04 (0.72, 5.81)	**0.4 (0.1, 0.5)**	**0.3 (0.1, 0.6)**	0.86 (0.29, 2.58)	**0.2 (0.1, 0.3)**	**0.2 (0.1, 0.2)**	1.03 (0.31, 3.38)	−0.0 (−0.2, 0.09)	−0.1 (−0.1, 0.2)
Cancer	0.90 (0.71, 1.12)	0.67 (0.40, 1.12)	**−0.4 (−0.4, −0.3)**	**−0.5 (−0.4, −0.6)**	0.87 (0.48, 1.59)	−0.1 (−0.1, 0.01)	−0.1 (−0.1, 0.1)	1.03 (0.53, 2.03)	**0.2 (0.2, 0.3)**	**0.2 (0.1, 0.4)**
Cardiovascular	**1.25 (1.08**, **1.44)**	0.83 (0.57, 1.21)	**−0.4 (−0.4, −0.3)**	**−0.4 (−0.3, −0.4)**	0.83 (0.55, 1.26)	**−0.4 (−0.4, −0.3)**	**−0.4 (−0.5, −0.4)**	0.98 (0.64, 1.51)	**0.1 (0.1, 0.1)**	**0.1 (0.1, 0.1)**
Chronic widespread pain	**1.54 (1.29**, **1.86)**	0.38 (0.21, 0.70)	**−1.3 (−1.3, −1.1)**	**−2.3 (−3.2, −1.6)**	0.75 (0.39, 1.41)	**−0.9 (−0.9, −0.8)**	**−1.1 (−1.5, −0.8)**	0.79 (0.38, 1.66)	**−0.6 (−0.6, −0.4)**	**−0.6 (−0.5, −0.8)**
Gastrointestinal	**1.34 (1.04**, **1.92)**	1.08 (0.67, 1.74)	**−0.2 (−0.2, −0.1)**	**−0.2 (−0.2, −0.1)**	1.04 (0.63, 1.73)	**−0.3 (−0.3, −0.2)**	**−0.3 (−0.3, −0.3)**	0.97 (0.57, 1.67)	**−0.2 (−0.3, −0.2)**	**−0.2 (−0.2, −0.2)**
Genitourinary	1.13 (0.70, 1.83)	1.09 (0.21, 5.71)	0.2 (−0.1, 0.2)	0.2 (−0.1, 0.7)	1.70 (0.35, 8.37)	0.2 (−0.1, 0.3)	0.2 (−0.1, 0.6)	1.61 (0.17, 14.73)	**0.6 (0.1, 1.0)**	**0.5 (0.1, 1.1)**
Endocrine	1.01 (0.84, 1.21)	0.75 (0.47, 1.21)	**−0.3 (−0.3, −0.2)**	**−0.3 (−0.3, −0.3)**	1.08 (0.64, 1.83)	**0.1 (0.1, 0.2)**	**0.1 (0.1, 0.2)**	1.27 (0.66, 2.44)	**0.5 (0.4, 0.7)**	**0.4 (0.3, 0.6)**
Musculoskeletal	1.01 (0.72, 1.41)	1.60 (0.81, 3.16)	**0.3 (0.2, 0.4)**	**0.3 (0.1, 0.5)**	0.85 (0.45, 1.62)	**−0.4 (−0.4, −0.3)**	**−0.4 (−0.3, −0.4)**	0.69 (0.37, 1.29)	**−0.6 (−0.6, −0.5)**	**−0.7 (−0.9, −0.5)**
Neurological	**1.32 (1.01**, **1.76)**	0.92 (0.48, 1.76)	**−0.3 (−0.4, −0.2)**	**−0.3 (−0.3, −0.3)**	0.70 (0.36, 1.38)	**−0.6 (−0.6, −0.5)**	**−0.7 (−0.5, −0.9)**	0.98 (0.45, 2.15)	**−0.1 (−0.3, −0.1)**	**−0.2 (−0.2, −0.1)**
Psychological	1.28 (0.86, 1.92)	0.43 (0.18, 1.04)	**−0.9 (−0.9, −0.7)**	**−1.5 (−2.2, −0.9)**	2.10 (0.67, 6.62)	**0.5 (0.1, 0.6)**	**0.3 (0.1, 0.7)**	0.81 (0.31, 2.15)	**−0.4 (−0.5, −0.2)**	**−0.4 (−0.4, −0.3)**
Respiratory	1.03 (0.78, 1.36)	1.25 (0.58, 2.67)	**0.2 (0.1, 0.3)**	**0.2 (0.1, 0.4)**	0.90 (0.44, 1.86)	**−0.2 (−0.3, −0.1)**	**−0.2 (−0.2, −0.1)**	0.66 (0.29, 1.48)	**−0.3 (−0.3, −0.2)**	**−0.3 (−0.3, −0.3)**

^a^ Adjusted for age, sex, BMI, remaining drugs and remaining comorbidities.

Bold indicates a value of *P* < 0.05.

KP: knee pain; RERI: relative excessive risk attributable to interaction.

**Fig. 2:**
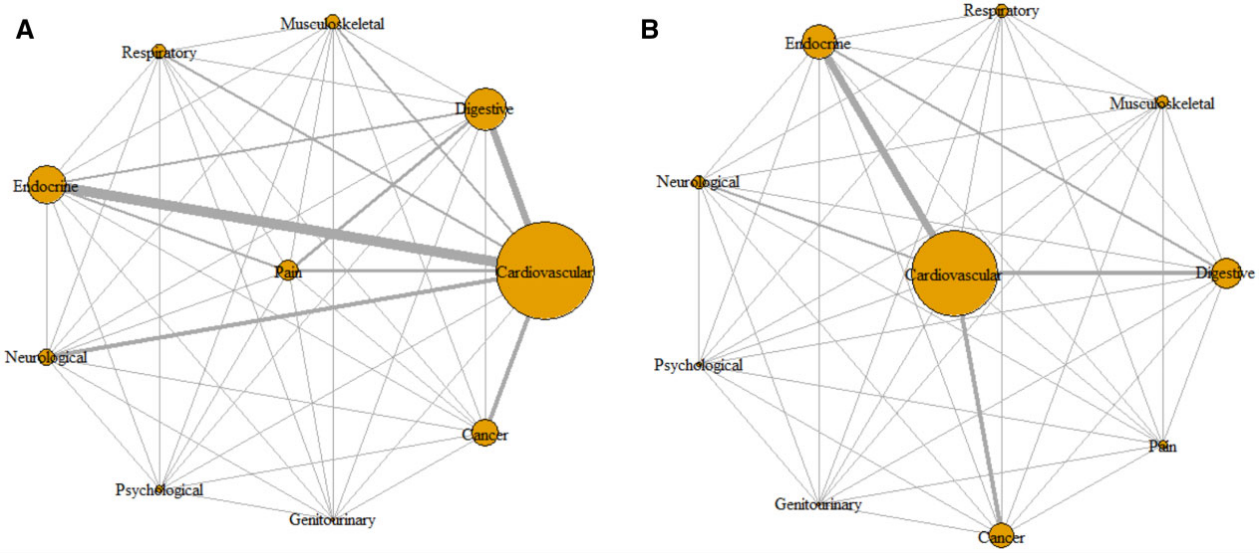
Comorbidity network in both the groups (**A**) Knee pain. (**B**) Non-knee pain. The different comorbidities are represented by nodes (circles); if there is a pairwise comorbid association in the cohort, the two disease nodes are connected with a line. The size of each node is proportional to the prevalence of the illustrated comorbidity, and the thickness of the line is proportional to the number of the pairwise associations. The nodes are placed such that those with more connections are closer to each other.

We plotted the pairwise association of comorbidities in both KP and non-KP groups separately (Fig. 2). The pattern of any two chronic conditions showed hypertension + high cholesterol, diabetes + hypertension, diabetes + high cholesterol and heart disease + high cholesterol to be the leading combinations in both groups (Supplementary Table S1, available at *Rheumatology Advances in Practice* online). However, the prevalence of the dyad patterns was always higher in the KP than the non-KP group (Supplementary Table S1 and Fig. S2, available at *Rheumatology Advances in Practice* online). KP had higher significant associations with each dyad pattern except for dyads that included cancer or stroke. The risk of having combined presence of IBS + FM and hypertension + FM was respectively 3.10 (95% CI 1.73, 5.55) and 3.49 (95% CI 1.88, 6.48) times higher in people with KP compared with non-KP (Supplementary Table S1, available at *Rheumatology Advances in Practice* online).

Examination of interactions of analgesic use with KP revealed no significant results in the multiplicative interaction model. However, in the additive interaction model, the interaction between paracetamol and KP contributes 30% (95% CI 8%, 60%) of risk for multimorbidity, 27% (95% CI 12%, 48%) of risk for musculoskeletal and 21% (95% CI 7%, 39%) of risk for respiratory conditions. For the remaining comorbidities, except genitourinary, the interaction of paracetamol with KP had negative contributions. Interaction between KP and opioids shared 18% (95% CI 1%, 20%) of the total association with the risk of multimorbidity, 34% (95% CI 8%, 70%) of association with psychological problems, and 8% (95% CI 2%, 19%) of association with endocrine disorders. Negative contributions were seen from opioids for the remaining comorbidities. The interaction between KP and NSAIDs contributed 22% (95% CI 12%, 39%) towards risk of cancer, 3% (95% CI 15, 11%) towards cardiovascular, 50% (95% CI 6%, 113%) towards genitourinary and 43% (95% CI 29%, 59%) towards endocrine disorders (Table 3). However, the association between NSAIDs and multimorbidity was not dose dependent according to the duration of use, whereas it was significant (*P* < 0.05) for opioids and paracetamol (Supplementary Fig. S3, available at *Rheumatology Advances in Practice* online).

## Discussion

The aims of this large, community-based study were to estimate the risk of comorbidities and the use of three types of oral analgesics in people with KP compared with those without. The key findings were that people with KP had greater risks of having multimorbidity, specifically chronic widespread pain, cardiovascular, gastrointestinal and neurological conditions than people without KP; and that although the use of paracetamol and opioids was increased, the use of NSAIDs was decreased in people with KP and comorbidities. The study has confirmed the current common use of oral analgesics (both prescribed and over-the-counter drugs) by people in the UK [32].

The increased prevalence of multimorbidity in people with OA and joint pain is increasingly evident in recent studies [33]. However, most of these studies have used electronic health records and have not clearly defined the site or nature of joint pain in community-based data, as we did in this study. Although we focused on KP as the symptom, the case definition also included self-reported physician diagnosis of OA. Multimorbidity patterns reported around the world have arthritis as one of the leading chronic conditions [34]. Multiple factors might be responsible for multimorbidity in KP and knee OA, such as age, BMI, pathology of the disease, physical inactivity and drugs used in pain management. The mean age of our study population was nearly 63 years, and we confirm a high prevalence of multiple chronic conditions in this age group [35].

Of the individual comorbidities, KP was most strongly associated with cardiovascular disease and diabetes. Likewise, in a study based in UK primary care, the risks of angina and heart failure were 36 and 28% higher, respectively, among OA cases compared with non-OA controls [25], and consistent results have been reported in other studies [36]. The relationship between chronic KP or OA and cardiovascular conditions can largely be explained through shared factors, such as higher BMI, ageing and reduced physical inactivity. We also found a strong association with high cholesterol in people with KP even after adjustment for age, sex, BMI and remaining comorbidities. High cholesterol, hypertension, obesity and diabetes are all components of metabolic syndrome, which is associated with KP [37]. Recent insights concerning chronic joint inflammation and its role in cardiovascular disease are relevant, and met-inflammation, adipokines and leptin might play a crucial role in explaining the linkage [38].

We also found strong associations with FM, fatigue and IBS. The associations with other chronic pain-related conditions, such as FM, back pain and IBS, could result from shared non-restorative sleep and central pain sensitization, which causes a decreased threshold to painful stimuli and exacerbates other causes of pain [39]. Estimates of the prevalence of chronic widespread pain in the general population vary from 1.4 to 24% [40]. Although KP is a regional presentation, chronic widespread pain in these people is augmented with symptoms of neuropathic-like pain [41]. The association reported in the present study could be an intersect of these two conditions that share mechanisms of central sensitization, but causation cannot be established in a cross-sectional study.

The risks of developing gastrointestinal problems, especially upper gastrointestinal problems such as gastritis and stomach ulcer, were highly associated with KP. Gastrointestinal disorders are known comorbidities in OA resulting from analgesic use [42]. We also found a positive association with vision problems in people with KP even after adjusting for ageing, high blood pressure and diabetes. More information is needed to study the temporal association and potential mechanisms between vision problems and KP, which is beyond the scope of this project.

A significant association of reported opioid use with KP and multimorbidity was seen in the study. Association of opioid prescription with multimorbidity is evident in both community and electronic health record research [43]. Opioids are prescribed drugs [44], although codeine is available over the counter in combination with paracetamol in the UK. The trend of opioid prescription has increased in recent years [32]. The association of opioid use in people with KP with multimorbidity or other specific comorbidities seen in the present study could be because comorbidities are associated with more severe KP and that comorbidities, such as chronic widespread pain, might encourage use of strong analgesics. Also, people with KP and comorbidities such as gastrointestinal and cardiovascular conditions, which contraindicate NSAIDs, instead might be more likely to be prescribed opioids or paracetamol [45]. The 34% attributed risk towards psychological problems observed in our study was similar to the Canadian population, where opioid use was associated with depression [46]. Paracetamol, the recommended first-line systemic analgesic for people with OA [2, 47], was also seen to be associated with comorbidities. The increased association of opioids and paracetamol with comorbidities needs an assessment regarding the consequence of this shifting, given the current evidence on the risk-to-benefit ratio of this first-line drug therapy for OA [2, 47].

Our interaction analyses showed that the use of NSAIDs is associated with an increase in risk for cardiovascular and genitourinary disorders, consistent with previous evidence of high risk of cardiovascular and renal problems with long-term use of NSAIDs [48]. Unlike most other studies, we used self-reported data from the community, which lacked detailed information on the prescriptions compared with specific recording in electronic health records but did include over-the-counter medicines. The negative interaction of NSAIDs and OA with comorbidities found in the present study accords with NICE advice [32] to prescribe NSAIDs with gastroprotective agents, and to not prescribe them to people with known gastrointestinal problems [47]. However, this association, with the potential of confounding by indication on this study, needs further investigation in large datasets.

There are several limitations to this study. Firstly, this is a cross-sectional study, hence it cannot explain the direction of the association (e.g. the use of analgesics and comorbidities might be bidirectional, and the explanation of the positive interaction for the increased use of paracetamol and opioids and negative interaction for the decreased use of NSAIDs needs further evidence to support any conclusions about causality). Secondly, the inherent recall bias in self-reported questionnaires might have under- or over-estimated the prevalence of the conditions and the use of medications. Thirdly, because of unavailability of complete data, we did not measure or adjust in our analyses for smoking status, education level or alcohol consumption, and these are important risk factors for comorbidities. Fourthly, we used KP and self-reported physician diagnosis of KOA, which is open to misclassification and ascertainment bias. As shown in Supplementary Fig. 1, available at *Rheumatology Advances in Practice* online, nearly half of the people in the KPIC survey did not have any information about KP status, which led to a reduced sample size and could have introduced selection bias.

### Conclusion

KP is associated with higher risks of multimorbidity and cardiovascular, chronic widespread pain, gastrointestinal and neurological comorbidities. There is a significant association of paracetamol and opioid use with comorbidities in people with KP compared with use of NSAIDs. Therefore, care must be taken, because the use of opioids and paracetamol instead of NSAIDs might still have severe consequences for older people with KP and other multiple chronic conditions. Further longitudinal large-scale studies are required to confirm the risk associations with opioids and paracetamol.

## Supplementary Material

rkac049_Supplementary_Data
