# Supplementary material for: Comorbidities and use of analgesics in people with knee pain: a study in the Nottingham Knee Pain and Health in the Community (KPIC) cohort
Source: Rheumatol Adv Pract. 2022 Jun 15;6(2):rkac049. doi: 10.1093/rap/rkac049 (PMC9245392; doi:10.1093/rap/rkac049)
Supplement: rkac049_Supplementary_Data [file rkac049_supplementary_data.zip › Appendix.docx]

**Appendices**

Appendix 1- Details of the conditions studied according to the groups

Appendix 2- Distribution of pattern of dyads (>=2 % prevalence) and the association with KP

**Appendix 1**- Details of the conditions studied according to the groups

| Groups (System specific) | Conditions included |
| --- | --- |
| Cardiovascular | Heart disease, Hypertension |
| Gastrointestinal | Liver and gall bladder disease, coeliac disease, colitis, upper gastrointestinal disorders |
| Chronic Widespread Pain (CWP) | Fibromyalgia, chronic fatigue syndrome, migraine, joint pain (excluding back pain and OA related), Irritable Bowel Syndrome |
| Musculoskeletal | Back problem, rheumatoid arthritis, gout, osteoporosis, psoriatic arthritis, lupus, polymyalgia |
| Neurological | Epilepsy, stroke, Parkinson, neurological (other) |
| Genito-urinary | Renal disease, Prostate problem |
| Psychological | Depression, Psychotic disorder, schizophrenia |
| Endocrine | Diabetes, Thyroid |
| Respiratory | Asthma, Chronic obstructive pulmonary disease (COPD) |
| Cancer | Any type |

**Appendix 2-** Calculation of attributable proportion due to addictive interaction (1)

We calculated the attributable proportion due to addictive interaction through following steps. (2)

If,

OR_11_- odds ratio for the group having both KP and medicine use

OR_10_- odds ratio for the group having KP and *no* medicine use and

OR_01_- odds ratio for the group having *no* KP and medicine use

OR_00_ – Reference group with odds ratio 1

**Step- 1**

Calculate respective relative risk (RR) from OR using the formula

RR_11_= OR_11_(1+OR_00_)/(1+OR_11_)

RR_10_= OR_10_(1+OR_00_)/(1+OR_10_)

RR_01_= OR_01_(1+OR_00_)/(1+OR0_1_)

**Step- 2** calculating the incremental effect otherwise commonly known as relative excess risk due to interaction (RERI) of analgesic on comorbidities in addition to KP was therefore calculated using the formula RR (RERI) = RR_11_-RR_10_-RR_01_+1.

An RERI value of 0 implies no additive interaction, whereas values greater than 0 imply superadditive (positive) interaction and values less than 0 imply subadditive (negative) interaction.

**Step 3**

Calculating the attributable proportion (AP) of risk due to interaction

AP = RERI/RR_11_

AP = 0 means no interaction or exactly additivity; AP > 0 means positive interaction or more than additivity; AP < 0 means negative interaction or less than additivity; AP can go from −1 to +1.

**Reference**

1. de Jager DJ, de Mutsert R, Jager KJ, Zoccali C, Dekker FW. Reporting of interaction. Nephron Clin Pract. 2011;119(2):c158-61. doi: 10.1159/000327598. Epub 2011 Jul 8. PMID: 21757954.

2. Kalilani L, Atashili J. Measuring additive interaction using odds ratios. Epidemiol Perspect Innov. 2006 Apr 18;3:5. doi: 10.1186/1742-5573-3-5. PMID: 16620385; PMCID: PMC1475799.
