# Supplementary material for: Comorbidities and use of analgesics in people with knee pain: a study in the Nottingham Knee Pain and Health in the Community (KPIC) cohort
Source: Rheumatol Adv Pract. 2022 Jun 15;6(2):rkac049. doi: 10.1093/rap/rkac049 (PMC9245392; doi:10.1093/rap/rkac049)
Supplement: rkac049_Supplementary_Data [file rkac049_supplementary_data.zip › 22-042 Supplementary Material.docx]

**Supplementary files**

Supplementary Figure S1- Flow Diagram of the participants in the study

Supplementary Figure S2- Dyad pattern of combination of chronic conditions in KP

Supplementary Figure S3- Dose response relationship between analgesics and multimorbidity stratified by KP status

Supplementary Table S1- Distribution of pattern of dyads (>=2 % prevalence) and the association with KP

Supplementary Figure S1- Flow Diagram of the participants in the study

Knee pain only= 1654

Knee OA only=272

Both Knee pain and Knee OA=906

Total KPIC Participants invited = 40,505

Total KPIC Participants data available = 9506

Participants available for study= 9428

Missing data on

Knee pain/ Knee OA= 4078

Knee pain/Knee OA= 2832

Non Knee pain or Knee OA=2518

Missing data either on Age or sex = 78

Participants available for study= 5350

Supplementary Figure S2- Dyad pattern of combination of chronic conditions in KP


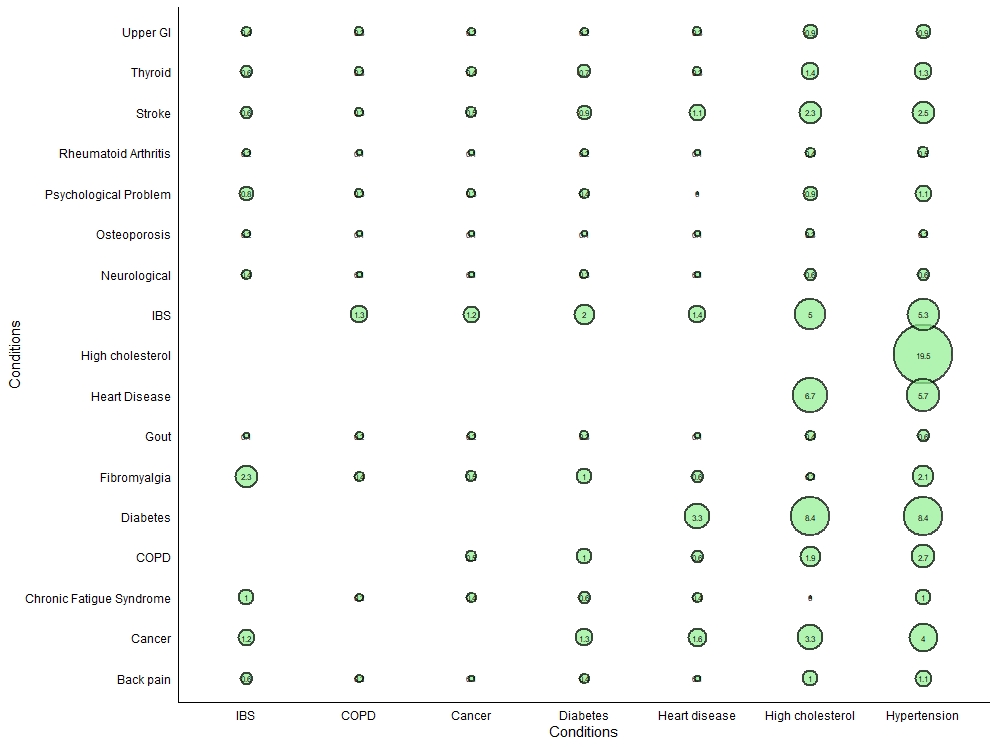


COPD- Chronic obstructive pulmonary disease; GI- Gastrointestinal; IBS- Irritable bowel syndrome

Conditions with >=1% prevalence is included in the figure.

The bubble size represents the proportionate prevalence of the dyad combination (row and column diseases) in each group.

Supplementary Table S1- distribution of pattern of dyads (>=2 % prevalence) and the association with KP

| Dyad Pattern | KP (n=2832) | Non-KP (n=2518) | Unadjusted OR (95% CI) | aOR  (95% CI) | |
| --- | --- | --- | --- | --- | --- |
|  |  |  |  | Model 1^$^ | Model 2^$$^ |
| High cholesterol + Hypertension | 552 (19.5) | 363 (14.4) | **1.44 (1.24-1.66)** | **1.37 (1.18-1.60)** | **1.26 (1.08-1.47)** |
| Diabetes + Hypertension | 238 (8.4) | 134 (5.3) | **1.63 (1.31-2.03)** | **1.58 (1.26-1.97)** | **1.05 (1.04-1.06)** |
| Diabetes + High Cholesterol | 238 (8.4) | 149 (5.6) | **1.56 (1.25-1.93)** | **1.53 (1.23-1.90)** | **1.47 (1.17-1.84)** |
| Heart diseases + High cholesterol | 191 (6.7) | 114 (4.5) | **1.52 (1.20-1.93)** | **1.49 (1.17-1.91)** | **1.47 (1.14-1.89)** |
| Hypertension + IBS | 151 (5.3) | 69 (2.7) | **1.99 (1.49-2.67)** | **1.87 (1.40-2.51)** | **1.69 (1.25-2.28)** |
| Hypertension + Cancer | 114 (4.0) | 82 (3.2) | 1.25 (0.93-1.66) | 1.12 (0.83-1.50) | 1.05 (0.77-1.43) |
| High cholesterol + Cancer | 94 (3.3) | 67 (2.6) | 1.26 (0.91-1.72) | 1.13 (0.82-1.56) | 1.04 (0.75-1.46) |
| COPD + Hypertension | 77 (2.7) | 30 (1.2) | **2.31 (1.51-3.54)** | **2.18 (1.42-3.34)** | **1.97 (1.27-3.06)** |
| Stroke+ Hypertension | 72 (2.5) | 37 (1.5) | 1.75 (1.17-2.60) | 1.61 (1.07-2.41) | 1.52 (1.00-2.30) |
| Stroke+ High cholesterol | 65 (2.3) | 39 (1.6) | 1.49 (1.00-2.22) | 1.40 (0.94-2.10) | 1.35 (0.89-2.05) |
| Fibromyalgia + IBS | 66 (2.3) | 15 (0.6) | **3.98 (2.26-6.99)** | **4.09 (2.32-7.21)** | **3.10 (1.73-5.55)** |
| Fibromyalgia +Hypertension | 60 (2.1) | 13 (0.5) | **4.17 (2.28-7.61)** | **3.91 (2.13-7.16)** | **3.49 (1.88-6.48)** |
| IBS+ Diabetes | 56 (2.0) | 16 (0.6) | **3.15 (1.80-5.51)** | **3.11 (1.78-5.44)** | **2.85 (1.60-5.05)** |

COPD- Chronic Obstructive Pulmonary Diseases; IBS- Irritable Bowel Syndrome; KP- Knee pain,

aOR- Adjusted Odds Ratio, CI- Confidence Interval

^$^Adjusted for Age, Gender, BMI

^$$^Adjusted for Age, Gender, BMI, opioids, NSAIDs, paracetamol

Bold indicates *P*-value < 0.05 (adjusted for multiple testing using Benjamin-Hochberg method)

Supplementary Figure S3- Dose response relationship between analgesics and multimorbidity stratified by KP status


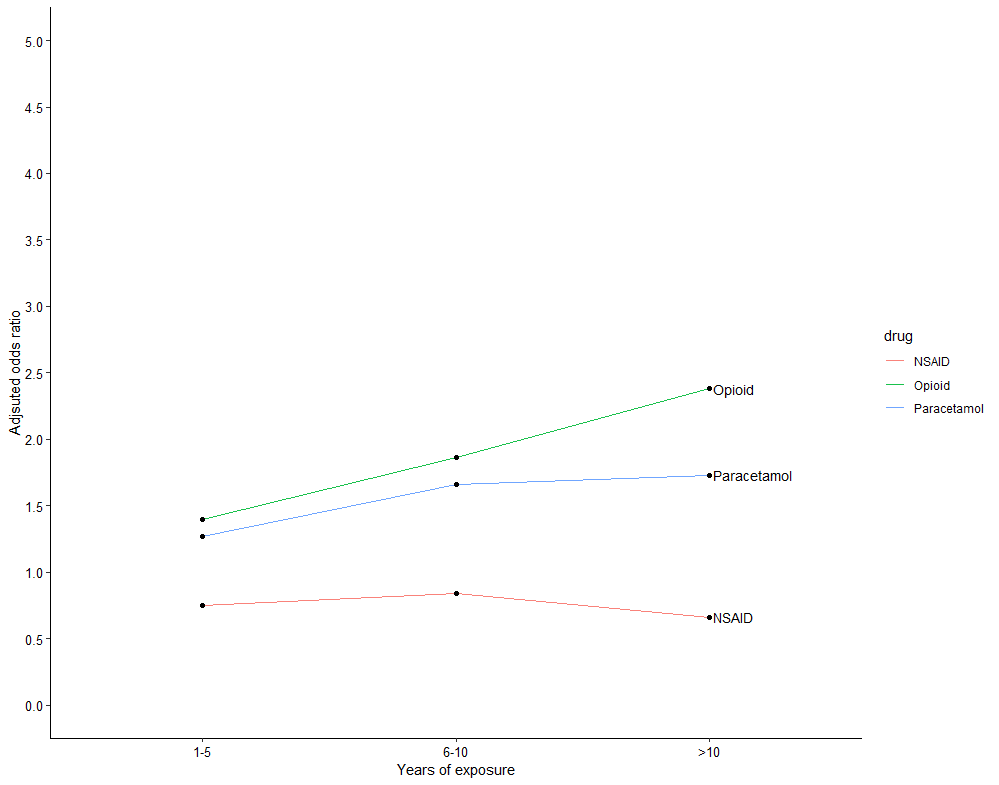


Trend test was significant for opioids and paracetamol at P value<0.05.
